# Supplementary material for: Implementation approaches for leprosy prevention with single-dose rifampicin: A support tool for decision making
Source: PLoS Negl Trop Dis. 2022 Oct 17;16(10):e0010792. doi: 10.1371/journal.pntd.0010792 (PMC9612816; doi:10.1371/journal.pntd.0010792)
Supplement: S1 SDR-PEP decision support tool — (PDF) [file pntd.0010792.s001.pdf]

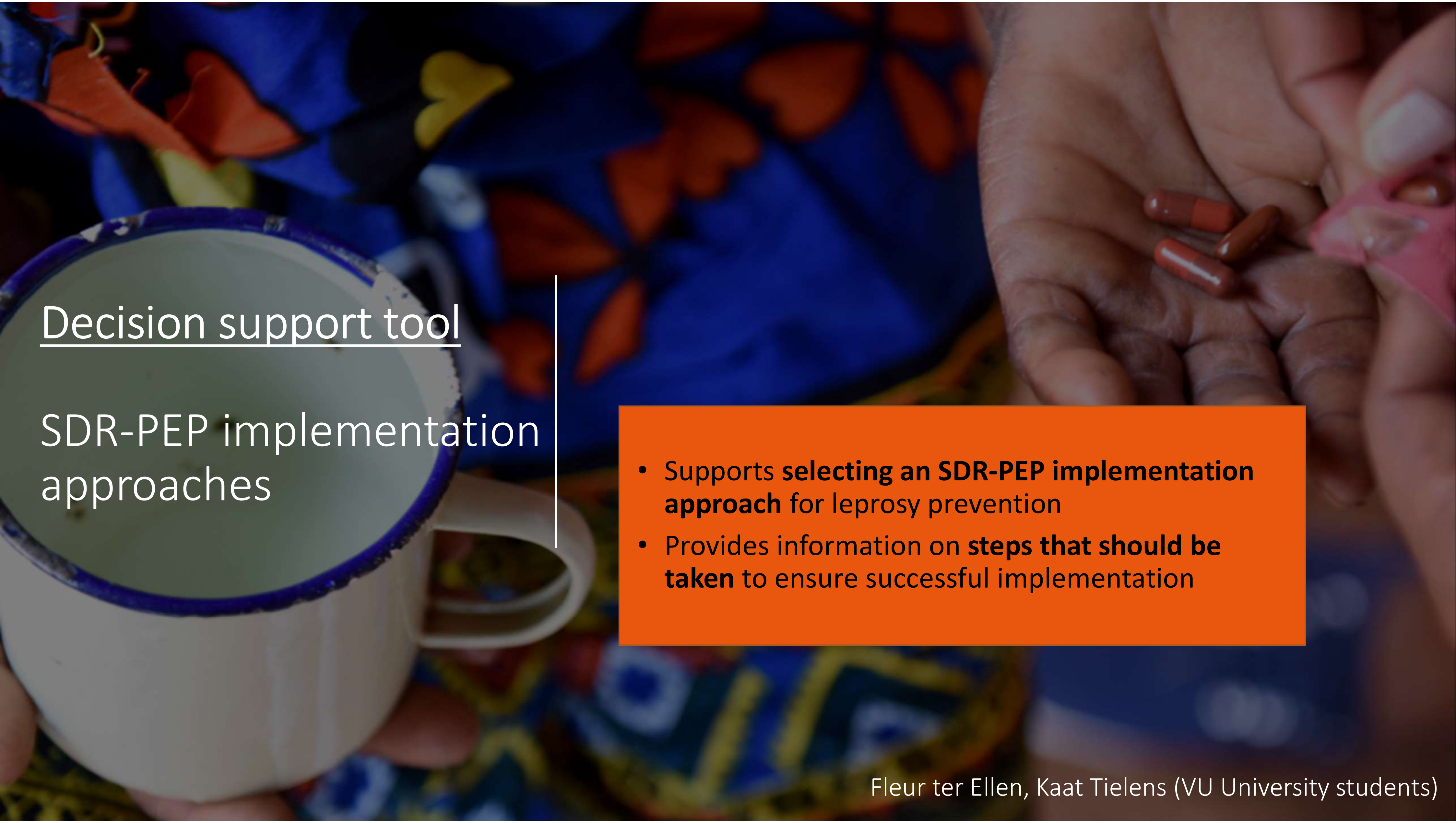

Decision support tool

SDR-PEP implementation approaches

- Supports **selecting an SDR-PEP implementation approach** for leprosy prevention
- Provides information on **steps that should be taken** to ensure successful implementation

Fleur ter Ellen, Kaat Tielens (VU University students)

What does this tool consist of?

- Description of implementation approaches
- Checklist basic requirements for successful implementation of any approach
- Flowchart to select implementation approach(es)
- Table with characteristics of the implementation approaches
- Annexes with reference material

Description of implementation approaches

A1. (Standard) close-contact approach

- Close contacts of new leprosy patients will be screened and given SDR-PEP when eligible.
- Close contacts are usually referred to as 20 of the closest contacts of an index patient; household contacts, neighbours and social contact (the number of contacts varies).
- Can be implemented through house-to-house visits, or contacts can be requested to come to a public location or health facility.

A.2. Non-disclosure close contact approach

- Close contact approach without disclosing the disease status of the leprosy patient by saying that there is leprosy in the area.
- May be required when disclosure is refused by the leprosy patient or in areas with high stigma levels.

B. Self-screening approach

- People will screen themselves and their household members for signs or symptoms of leprosy with guidance from an instruction form.
- When leprosy is suspected, a health worker should be contacted who could screen the household members again.
- This way people become more aware of signs and symptoms of leprosy and more contacts per index patient can be covered.

Description of implementation approaches

C.1. Blanket approach / mass drug administration

- An entire population will be screened for leprosy and receive SDR-PEP when eligible.
- This approach is resource intensive and requires thorough preparation.
- Recommended for highly endemic settings and found to be suitable for areas that are hard to reach.

C.2. Mass drug administration in clusters / focal mass drug administration (fMDA)

- Mapping, and geospatial analysis should be done first to identify the clusters for implementation.
- This approach can be combined with self-screening and/or serology.
- This approach is resource intensive and requires thorough preparation.
- This approach to administer SDR-PEP has not been piloted yet.

Description of implementation approaches

D. Retrospective-active case finding campaign/drives

- All leprosy cases diagnosed in a pre-defined time-period are traced and SDR-PEP is administered to their eligible contacts.
- This can be organised in so-called ‘drives’, performed by mobile teams of leprosy experts.
- Availability of an accurate database of leprosy patients is required and comprehensive logistic preparation.

E. Skin camp / Community based approach

- Community health camps will be set up where people will be screened for multiple skin diseases, including leprosy (integrated skin screening).
- Health workers in mobile teams execute these skin camps, in collaboration with community volunteers and preferably a dermatologist.
- SDR-PEP will be administered to all eligible persons that are attending the skin camp.
- Skin camps have not been specifically used for SDR-PEP administration before, but the feasibility of this approach is currently being studied.

How to use this tool?

Step 1	Check whether all basic requirements are met to ensure successful implementation of SDR-PEP.
Step 2	Make use of the flowchart to select the most appropriate approach(es). Use your knowledge of the area to fill in the flowchart.
Step 3	Check the additional information about the selected approaches (based on evidence and lessons learned from countries with SDR-PEP implementation experience).

1. Checklist basic requirements for the successful implementation of any approach

Aspect	Requirements	Is/can the requirement be met?
Support	Governmental commitment to ensure sustainable SDR-PEP implementation	Yes/No
	Financial and technical support	Yes/No
	Involvement of persons affected by leprosy	Yes/No
	Support from health staff and community volunteers	Yes/No
	An overview of stakeholders	Yes/No
Medication (rifampicin, MDT)	Governmental support to procure rifampicin	Yes/No
	Availability of sufficient rifampicin and MDT	Yes/No
Health system	A surveillance system adapted to SDR-PEP implementation*	Yes/No
	Thorough understanding of required communication, organisation and implementation of the approach at the various levels of the health system	Yes/No
Trained health staff	Sufficient trained health staff is (or will be) available	Yes/No
Health education	Health education & community awareness raising in the targeted area	Yes/No

* For more information about the minimal set of data required to appropriately document contact tracing activities and SDR-PEP administration: Richardus et al. (2018) [1]

2. Flowchart

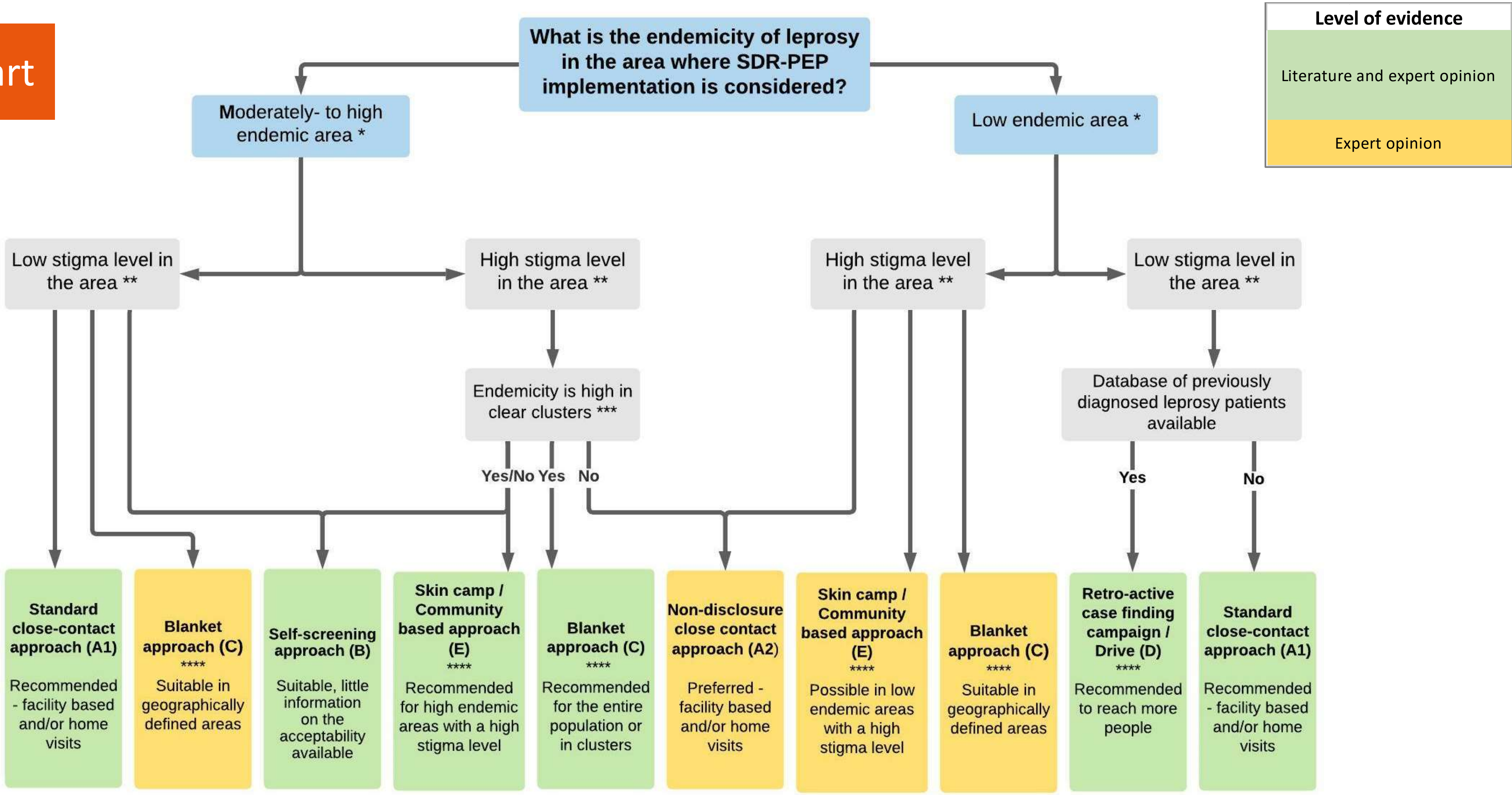

* Need help to determine the level of endemicity in the targetted area? See Annex I

** Need help to determine the level of stigma in the targetted area? Seen Annex II

*** Mapping of patients is advised to identify clusters

**** When an area is hard to reach and/or geographically isolated, an approach aimed at reaching a high number of people is preferred to make maximum use of the logistics involved, like: Blanket approach, Skin camp / Community approach or Retro-active case finding / Drive.

Level of evidence
Literature and expert opinion
Expert opinion
Literature

3. Characteristics of the approaches: obtained from literature/experts

Characteristics of the chosen SDR-PEP implementation approach(es):		A. Close contact approach		B. Self-screening approach	C. Blanket approach		D. Retro active case finding (RACF) / Drives	E. Skin camp / Community based approach (pilots in initial stages)
		A.1. (Standard) close contact approach	A.2. Non-disclosure approach		C.1. Mass drug administration to entire population	C.2. Mass drug administration in clusters (not tested yet)		
**Acceptance** of stakeholders	*High / low*	**High**	**High**	**No information**	**High**	**Expected to be high**	**High**	**High**
**Targeted contacts**	*Household*	**Yes**	**Yes**	**Yes**	**Yes**	**Yes**	**Yes**	**Yes**
	Neighbours	**Yes**	**Yes**	**Yes**	**Yes**	**Yes**	**Yes**	**Yes**
	Social contacts	**Yes/No**	**Yes/No**	**Yes/No**	**Yes**	**Yes**	**Yes/No**	**Yes**
	Community	**No**	**Yes/No**	**Yes/No**	**Yes**	**Yes**	**No**	**Yes**
**Costs**	*High / low / cost-effective*	**Cost-effective for all contacts**		**Low**	**High**	**No information**	**No information**	**High**
**Human resources**	*Medical staff / community health workers / volunteers*	Medical staff required Community health workers and volunteers could help with screening	Medical staff required Community health workers and volunteers could help with screening	Medical staff required for confirmation or exclusion of leprosy	Medical staff required with support from community health workers	Medical staff required with support from community health workers	Medical staff required, leprologist or dermatologist if available	Medical staff required, preferably a dermatologist Community health workers and volunteers could help organising
**Logistical preperations**	*Specifics for the approach*	Door-to-door visits require more resources and preparations than a facility-based approach		Self-screening forms containing pictures and text aids to self-check for signs of leprosy	A few resource intensive visits	Mapping, and geospatial analysis should be used to identify high endemic areas	Requires accurate register of leprosy patients	Requires good referral system

Annex I - Endemicity

Definitions of endemicity (*)

**High endemic**	NCDR > 100 new autochthonous cases per 1 million population per year
**Moderately endemic**	NCDR 50 to 100 new autochthonous cases per 1 million population per year
**Low endemic**	NCRD <50 new autochthonous cases per 1 million population per year
**Non endemic**	No autochthonous cases for at least 10 years

NCDR: New case detection rate

(*) Based on the report of the WHO Task Force on definitions, criteria and indicators for interruption of transmission and elimination of leprosy, March 2021 [2]

Annex II – Stigma assessment

Easy to use, free and accessible stigma measurement tools that can support the assessment of stigma levels are summarized below.

Stigma measurement tools	Information	Strenghts	Limitations
5-Question Stigma Indicators	5-item questionnaire available in two versions: (1) community-based version, (2) version that could be assessed by persons affected by leprosy.	Expected easy to use, recommended in WHO's Monitoring and Evaluation Guide	Validation took only place in India.
EMIC affected persons	15-item questionnaire for persons affected by leprosy that evaluates the experienced as well as the perceived stigma.	Adapted in different languages and validated for different countries.	Tool could not be used by program staff and community members.
EMIC-CS community stigma	15-item questionnaire that measures perceived behaviour and attitudes towards persons affected by leprosy, their family and leprosy in general.	Adapted in different languages and validated for different countries.	Tool does not involve persons affected by leprosy.
SDS Social Distance Scale	7-item questionnaire that measures the attitudes towards different social relationships with a person affected by leprosy by using a vignette that describes a person affected by leprosy.	Short and easy to use.	Tool does not involve persons affected by leprosy.
Participation Scale Short	13-item short version of the Participation Scale that measures the severity of the participation restrictions from the perspectives of persons affected by leprosy.	Available in at least 25 languages.	Additional field testing is needed to confirm the promising results on its validity.

More information? The *Guides on Stigma and Mental Wellbeing*, especially Guide 4, provide a comprehensive overview of stigma assessment methods and tools [3]. The flowchart in Guide 4, Annex 3 could be used to select a suitable assessment method to assess the stigma level.

Annex III – Recommended documents to consult

World Health Organization:

- World Health Organization. (2021). Towards zero leprosy. Global leprosy (Hansen's Disease) strategy 2021–2030.
- World Health Organization. (2020). Leprosy/Hansen disease: Contact tracing and post-exposure prophylaxis.
- World Health Organization. (2018). Guidelines for the diagnosis, treatment and prevention of leprosy.

Research:

- Moet, F. J., Pahan, D., Oskam, L., & Richardus, J. H. (2008). Effectiveness of single dose rifampicin in preventing leprosy in close contacts of patients with newly diagnosed leprosy: cluster randomised controlled trial. Bmj, 336(7647), 761-764.
- Richardus, J. H., Tiwari, A., Barth-Jaeggi, T., Arif, M. A., Banstola, N. L., Baskota, R., ... & Steinmann, P. (2021). Leprosy post-exposure prophylaxis with single-dose rifampicin (LPEP): an international feasibility programme. The Lancet Global Health, 9(1), e81-e90.

References:

1. Richardus JH, Kasang C, Mieras L, Anand S, Bonenberger M, Ignotti E, et al. Minimal essential data to document contact tracing and single dose rifampicin (SDR) for leprosy control in routine settings: A practical guide. Lepr Rev. 2018;89(1):2–12.
2. WHO. Task Force on definitions, criteria and indicators for interruption of transmission and elimination of leprosy: Report of the final meeting March, 2021
3. ILEP, NNN. Guides on Stigma and Mental Wellbeing. June 11th, 2020

Take a step to implement SDR-PEP

https://youtu.be/uC-ADLv4SPM

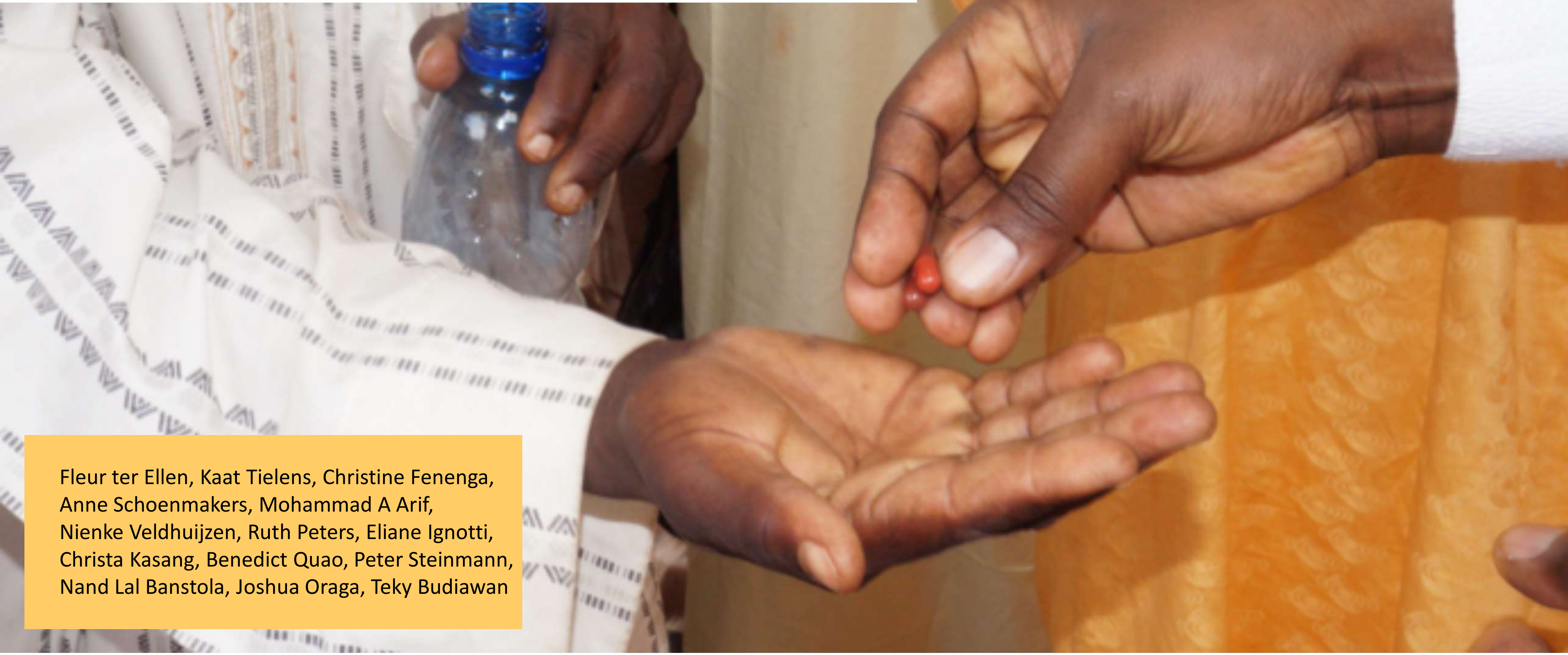

Fleur ter Ellen, Kaat Tielens, Christine Fenenga,
Anne Schoenmakers, Mohammad A Arif,
Nienke Veldhuijzen, Ruth Peters, Eliane Ignotti,
Christa Kasang, Benedict Quao, Peter Steinmann,
Nand Lal Banstola, Joshua Oraga, Teky Budiawan
