# Supplementary material for: Implementation approaches for leprosy prevention with single-dose rifampicin: A support tool for decision making
Source: PLoS Negl Trop Dis. 2022 Oct 17;16(10):e0010792. doi: 10.1371/journal.pntd.0010792 (PMC9612816; doi:10.1371/journal.pntd.0010792)
Supplement: Attachment — Submitted filename: Review note PLOS2.pdf [file pntd.0010792.s004.pdf]

Section	Review comments and notes
Abstract	- Please check the broken link in the abtract
Introduction/ background	- It clear
Methods	- In Phase !. Literature review, please describe the search timeline of articles published (is it May to June 2020?) (page 7) because in the result the author mentioned that articles were published between 2005-2020 (page 10)
Results	- The result is more readable than the previous version.<br>- Please do not put any comments, interpretations or references in the result, put them into the discussion (check page 14-27)<br>- Where I and the reader can find the SDR-PEP decision support tool?
Discussion and Conclusions	- Do not mix between result and discusiion
Overall	-The paper is more readable than the previous version, the author already put the additional table, and figures to make the article more readable.<br>-It is still confusing between the result and discussion, the author mixed it<br>-Please provide the tool in the manuscript or link because it can not find in the link or in pdf
