# Supplementary material for: Implementation approaches for leprosy prevention with single-dose rifampicin: A support tool for decision making
Source: PLoS Negl Trop Dis. 2022 Oct 17;16(10):e0010792. doi: 10.1371/journal.pntd.0010792 (PMC9612816; doi:10.1371/journal.pntd.0010792)
Supplement: Attachment — Submitted filename: Review note PLOS2.docx [file pntd.0010792.s002.docx]

| **Section** | **Review comments and notes** |
| --- | --- |
| Introduction/  background | - The author clearly define the situation related the topic in the introduction but there is statement that confusing in Alinea 3..” this study proved… 57%’.. this statements should not appear in the introduction. - The number 57% also did not support data in the result of the manuscript. - Please arrange the introduction section so that easy to understand, the author, one paragraph one idea or topic |
| Methods | - The study consisted two phases,the first the development process with the literature review, the second was evaluated the usability and applicability with FGD and interview. - The first phase was described the query method and expert interviews. In the expert interviews authors were not clearly define how many experts, who etc. but it put in the result, while in the results are not given anything as the result of the process, same with the literature review in the result. - If the process was complex, please put a diagram to describe it. |
| Results | - Please arrange the results in way the author described in the methods consist of phase 1 an phase 2. - The reader can not find the results of the study easily such as what is the result from literature review? Expert interview? And so on.. its confusing - Add some tables as conclusion from all of the result in each phase don’t mix the result of each phase - Add table result from: literature review, FGD and interview with thematic analysis - The reader difficult to find the meaningful result. The author should put the result in the manuscript not only link. Please choose the most meaningful result. - Do not put any references in the result.. just describe the result in each phases clear and easy to understand eventhough the study was conducted qualitative methods in collecting data. |
| Discussion and Conclusions | - Please do not discuss any thing that not in the result. Author mention percentage numbers that not related in the manuscript. - In the manuscript should give the details in SDR-PEP decision support tool so easy to discuss each component in it. - Please arrange the discussion related the important result |
| Overall | The manuscript is to abstract and missing the important things that should describe, the author should include the important result in the manuscript not to ask the reader to open many links |
