# Supplementary material for: Implementation approaches for leprosy prevention with single-dose rifampicin: A support tool for decision making
Source: PLoS Negl Trop Dis. 2022 Oct 17;16(10):e0010792. doi: 10.1371/journal.pntd.0010792 (PMC9612816; doi:10.1371/journal.pntd.0010792)
Supplement: Attachment — Submitted filename: 220604 Responses to reviewer comments.docx [file pntd.0010792.s003.docx]

**04-06-2022**

**Dear PLOS-NTD editors,**

Thank you for the valuable input you shared with us. We believe to have addressed your points, which has led to an improved manuscript.

Before we provide you with our response to the reviewers’ comments, please allow me to address the point you raised in your email:

*In addition to the recommendations and suggestions indicated by the reviewers, concern arose regarding the “Decision tool SDR-PEP implementation” tool, since it is available on the website of the organization Infolep – Leprosy information services, as per the link below:* [*https://linkprotect.cudasvc.com/url?a=https%3a%2f%2fwww.leprosy-information.org%2fresource%2fdecision-tool-sdr-pep-implementation.&c=E,1,M9D3lEzO4T8F5jmvgtiqIIT3ra6tKZff_YLPD9y3cnwcMQ-YBNBPbdNuwxxl-vXqWaNig8yBA0elVmO9141DMm_F2hbi6FSsDaWKtus6Tr8,&typo=1*](https://linkprotect.cudasvc.com/url?a=https%3a%2f%2fwww.leprosy-information.org%2fresource%2fdecision-tool-sdr-pep-implementation.&c=E,1,M9D3lEzO4T8F5jmvgtiqIIT3ra6tKZff_YLPD9y3cnwcMQ-YBNBPbdNuwxxl-vXqWaNig8yBA0elVmO9141DMm_F2hbi6FSsDaWKtus6Tr8,&typo=1)

*Please clarify this situation.*

- *The tool was placed on the online portal (Infolep) for ease of reference, so that you would have it ready at hand. It was only available for those who had received the link. We have now removed it from the Infolep portal and have uploaded a digital copy in the PLOS-NTD portal.*

You will find our response to your input below and we have shared an adapted manuscript: one with track-changes and one without. The line numbers mentioned relate to the manuscript with track-changes.

<b>Methods</b></br></br>

-Are the objectives of the study clearly articulated with a clear testable hypothesis stated?</br> -Is the study design appropriate to address the stated objectives?</br> -Is the population clearly described and appropriate for the hypothesis being tested?</br> -Is the sample size sufficient to ensure adequate power to address the hypothesis being tested?</br> -Were correct statistical analysis used to support conclusions?</br> -Are there concerns about ethical or regulatory requirements being met?</br></br>

Reviewer #1: This study is based on eliciting opinions about how best to implement post-exposure prophylaxis (PEP) as a preventive measure against leprosy.

Two groups of interviewees are described, one consisting of 13 individuals with prior knowledge of PEP implementation and the other of 15 individuals with little or no previous experience of PEP.

There are 15 authors of the paper, but their roles are not clearly described, although in the text 6 of the authors are said to have undertaken certain tasks.

In my opinion, there is a need for greater clarity about who did what in this study. What were the roles of each of the co-authors? How were the two groups of participants selected? Who carried out the interviews and FGDs? Were any of the authors included in the list of experts?

*Thank you, we hope we have now better clarified the role of the co-authors by adding additional information about who contributed to what during the research and manuscript writing (line numbers 228-229). This is addressed in more detail in the text and under the methodology – ethic section (line numbers 164-169). The idea for this study arose during a PEP research workshop. This is now explained in the introduction (line numbers 131-134). Several co-authors took part in this meeting. Some of them were interviewed as experts (also described in line numbers 131-134, 254).*

There is very little information about exactly how the 5 types of PEP implementation were identified - it was simply "based on the review and expert interviews". One might imagine that certain tentative conclusions were drawn after the literature review, and the list was then refined through the interviews; more transparency about this process would be welcome.

*Thank you for your comment. We agree that this needs to be more clear. Additional text on the results of the literature review and expert interviews is added. Also, a table (2) indicating which sources were used to identify the SDR-PEP implementation approaches and basic requirements (literature and experts) is inserted (line number 284-285).*

*A figure (3) from the tool is also included, which indicates whether information on characteristics of the approaches is based on literature, expert opinion, or both (line number 286-287).*

Reviewer #2: The limitation of articles consulted is quite understandable, since this WHO recommendation is still not very widespread.

This restriction may also have influenced the health professionals selection to be interviewed

However, the tool created should be useful to add-on WHO guideline for leprosy contacts tracing in SDR-PEP.

*Yes, indeed. This study was set-up to develop a practical tool as an add-on to WHO guidelines.*

Reviewer #3: I consider this an important and timely publication in support of the implementation of novel preventive interventions in the field of leprosy. It is very well written and provides a wealth of information about the state of the art of SDR knowledge and experience, as well as very detailed and practical guidelines (the support tool) for deciding on appropriate strategies in given settings.

The publication is based on a mixed methods approach, including literature review, expert interviews and focus group discussions. I particular like the classification of close contact survey in various alternative approaches, which on one hand clarifies the different possibilities and certainly will help field programs to choose the most appropriate and feasible approach in their own setting. The description of basic requirements for implementation is also very informative and useful.

The focus of this publication is on the decision support tool that is based on the studies. A strong feature of this paper is that it includes the development and evaluation of the tool with the intended end users. In that sense it represents a full cycle of research, development, evaluation and presentation of the final product; the tool.

*Thank you for your positive feedback.*

With regard to methodology, the only comment I have is that the literature review includes English language publications only. It may have been useful to include Portuguese, Spanish and French language publications as well? On the other hand, the interviews and focus groups included many people from endemic countries.

*Thank you.*

*Indeed, one of the limitations of this study is the Anglophone literature used only. This is now addressed in the discussion section (line number 658-659). However, by including experts from non-Anglophone countries in the study, we aimed to capture experiences and views from as many leprosy endemic countries as possible (table 1, line number 261).*

Reviewer #4: The study objectives are clear but no hypothesis was defined. It is important to define the hypothesis or the group assumption when developing this decision support tool about the SDR-PEP as well as evaluate its applicability.

*Thank you for your suggestion.*

*This study took a predominantly qualitative approach which does not require a 'hypothesis'. The study endpoint was a decision support tool, so the manuscript described the scientific basis for the development of this tool. During the PEP meeting with experts, we expected that through literature review and additional expert interviews we would be able to collect useful data for the development of a decision support tool. In addition, we expected that involving the end users of the decision tool in our study would increase the usability and applicability of the tool. These expectations are now described in more detail in the introduction in this new version of the manuscript (line number 149-151).*

Regarding the study design, the methodology is considered appropriate to support the defined goals.

*Thank you.*

The study population is clearly described, but I suggest moving part of the text that is placed in the results section as “Participants interviews and focus group” where this population had been better characterized, to the methods section. Also, in the methods section, in the paragraph that the participants of the second stage of the study are described.

*Thank you for your suggestion. We consider the information on the interviewees and FGD participants relevant for the result section. It indicates the variety of people interviewed and their backgrounds, which is part of and relevant for the interpretation of the findings.*

I suggest explaining more carefully that one of the participants was included only from the focused group.

*Thank you. We have adapted the text to indicate this more clearly (line number 512).*

About the compliance with ethical requirements, the number of the research ethics committee approval document may be inserted.

*Formal ethical approval was obtained, we inserted the number in the manuscript (line number	160)*

Reviewer #5: - The author clearly define the situation related the topic in the introduction but there is statement that confusing in Alinea 3..” this study proved… 57%’.. this statements should not appear in the introduction.

-	The number 57% also did not support data in the result of the manuscript.

*Thank you for your comment. The 57% relates to the effectiveness of SDR-PEP, which is based on the COLEP study as indicated in the text (see reference list). We believe this information is relevant information for the reader who has no prior knowledge regarding SDR-PEP. It is not a research finding in this study; it is background information on SDR-PEP.*

-	Please arrange the introduction section so that easy to understand, the author, one paragraph one idea or topic

*Thank you for your comment. We removed some detailed information from the introduction to increase the readability (line number 120-123)*

-	The study consisted two phases, the first the development process with the literature review, the second was evaluated the usability and applicability with FGD and interview.

-	The first phase was described the query method and expert interviews. In the expert interviews authors were not clearly define how many experts, who etc. but it put in the result, while in the results are not given anything as the result of the process, same with the literature review in the result.

*Thank you for this comment. It overlaps with a comment made by reviewer one and we agree that these results need to be more clearly addressed.*

*We have therefor inserted additional text on the results of the literature review and expert interviews (line number 267-279). In addition, a table (2) indicating which sources were used to identify the SDR-PEP implementation approaches and basic requirements (literature and experts) is inserted (line number 284-285). In addition, a figure (3) from the tool is included, which indicates whether information on characteristics of the approaches is based on literature, expert opinion or both (line number 286-287).*

If the process was complex, please put a diagram to describe it.

*Thank you for the suggestion. We have inserted a figure (1) that provides a visual overview of the methodology (line number 154-156). We have also re-structured the document more clearly into the two phases, which should increase its readability and decrease the impression of complexity.*

--------------------

<b>Results</b></br></br>

-Does the analysis presented match the analysis plan?</br> -Are the results clearly and completely presented?</br> -Are the figures (Tables, Images) of sufficient quality for clarity?</br></br>

Reviewer #1: Greater transparency in how the list of five types was generated is needed.

*Thank you, we agree with this comment and have addressed this by inserting more information on the (process of obtaining) results in the text and by including a table (2) and two figures (1 and 3) that give more detailed information on how the list was generated.*

Reviewer #2: The authors statement related to the main limitations of the study are satisfactory and the discussion adequately connected the research objectives.

*Thank you*

Reviewer #3: The results are clearly presented. In particular the tool itself, the main result, is very clear and accessible, with much detail.

*Thank you*

Reviewer #4: In the results section – literature review, check the information related to the correct number of articles excluded after reading the full text. The flowchart states that 55 articles were excluded, which is consistent with the final number included in the review (33). However in the text body 52 articles are shown.

*Thank you for your observation. The higher number of articles is due to the fact that additional articles are referred to in the introduction and discussion section and are therefore part of the reference list of the article.*

In the literature review, the main information extracted from the selected articles as well as the summarized data of these studies were not presented. I suggest the inclusion of a summary table as supplementary material. Throughout the text it is described that the data were extracted from the articles using a data extraction form. It defines what kind of information was cited such as country, type of approach and characteristics, context, stated pros and cons, resources required, and acceptability. However it does not show the extracted information, nor to which articles each information corresponds.

*Thank you for your comment. The additional table (2) and figures ( 1 and 3) should provide a better insight in the sources used for the data extracted. Also, the data extraction form and topic list have been uploaded for ease of reference.*

In addition, in the part that reports the interviews with experts, it was described that the collected information from the interviews were also used to develop the tool, but it does not make it clear which data were collected during the interview and which ones were important in its development. Besides, what are the guiding questions of these interviews? It is also necessary to explain the technique used to construct the five categories.

*Thank you for your comment. We now added the aim of the interviews and the topics that were discussed with the experts to the method section of the manuscript (line number 200-203). We also added the data extraction forms for the literature review and the interview topic list.*

Some results, such as the one presented in the item “C.2. Blanket approach / mass drug administration in clusters” do not refer to any study in the literature review nor to the speech of any specialist who supports the definition of this category as presented for the other categories. Thus, it difficult to understand the emergence of this category among the five approaches considered to be the main ones in the formulation of the tool.

*Thank you for your comment. It is a sub-category of the ‘blanket approach’. This approach was indeed added by the experts, not extracted from the literature. The newly inserted table (2) clarifies the sources used for each of the approaches (line number 284-285).*

In general, the results have already been presented grouped in the approaches defined in the tool, mixing what was found in the literature with the speech of the specialists. Greater clarity of these data is important, as they support the conclusion of the study.

*Thank you for your comment.*

*Table 2. is added to the result section to clarify the sources on which the SDR-PEP approaches are based (line number 284-285).*

Reviewer #5: - Please arrange the results in way the author described in the methods consist of phase 1 an phase 2.

-	The reader can not find the results of the study easily such as what is the result from literature review? Expert interview? And so on.. its confusing

-	Add some tables as conclusion from all of the result in each phase don’t mix the result of each phase

-	Add table result from: literature review, FGD and interview with thematic analysis

-	The reader difficult to find the meaningful result. The author should put the result in the manuscript not only link. Please choose the most meaningful result.

-	Do not put any references in the results. just describe the result in each phase clear and easy to understand even though the study was conducted qualitative methods in collecting data.

*Thank you for the suggestions. We have restructured the manuscript to increase its readability and reduce confusion. Additional tables/figures should help the reader to understand the study process. We also changed the order of the results section and tried to organize it more in line with the research phases. The references in the results section, directly refer to the literature found in the review.*

--------------------

<b>Conclusions</b></br></br>

-Are the conclusions supported by the data presented?</br> -Are the limitations of analysis clearly described?</br> -Do the authors discuss how these data can be helpful to advance our understanding of the topic under study?</br> -Is public health relevance addressed?</br></br>

Reviewer #1: The final list is helpful.

*Thank you.*

Reviewer #2: Yes, it is relevant in addressing a current WHO recommendation and fills in the lack of studies on this subject

*Thank you.*

Reviewer #3: The conclusions are to the point. It also points out areas for further development of the tool. Costing aspects and cost-effectiveness analysis of SDR-implementation approaches is certainly a next step.

*Thank you.*

Reviewer #4: The study conclusion is clear and supported by the data presented throughout the article and in the document “Decision tool SDR-PEP implementation”, highlighting the importance of the developed tool. In addition, it responds to the proposed objectives. The limitation of the developed tool was described, as well as the need for new studies that could overcome its limitations as new analyzes that would result in the improvement of the appliance.

*Thank you.*

Reviewer #5: - Please do not discuss anything that not in the result. Author mention percentage numbers that not related in the manuscript.

-	In the manuscript should give the details in SDR-PEP decision support tool so easy to discuss each component in it.

-	Please arrange the discussion related the important result and component in SDR-PEP decision support tool

*Thank you for your comment. The manuscript has improved based on the reviewers’ comments received. We believe however, that the discussion and conclusion are in support of the final result.*

--------------------

<b>Editorial and Data Presentation Modifications?</b></br><br/> Use this section for editorial suggestions as well as relatively minor modifications of existing data that would enhance clarity. If the only modifications needed are minor and/or editorial, you may wish to recommend “Minor Revision” or “Accept”.

Reviewer #1: (No Response)

Reviewer #2: There is still some resistance to the implementation of Chemoprophylaxis in the leprosy control programs. Therefore, perhaps it could be minimized. One suggestion is to highlight that the tool is useful not only for the SDR-PEP purpose, but also for immunoprophylaxis and early diagnosis (in the conclusion and in the abstract).

*Interesting suggestion, thank you. This study was quite specifically aimed at supporting SDR-PEP implementation. However, a sentence on its possible relevance for immunoprophylaxis has been added in the discussion section (line number 689-690).*

Reviewer #3: Well written report and very relevant for the field of leprosy. My advice is to accept.

*Thank you.*

Reviewer #4: (No Response)

Reviewer #5: The topic is interesting and important even though the manuscript should arrange to become readable

*We have changed the structure of the article, removed sections, and added some additional tables/figures to better clarify the study process and make the article easier to read.*

--------------------

<b>Summary and General Comments</b></br></br> Use this section to provide overall comments, discuss strengths/weaknesses of the study, novelty, significance, general execution and scholarship. You may also include additional comments for the author, including concerns about dual publication, research ethics, or publication ethics. If requesting major revision, please articulate the new experiments that are needed.

Reviewer #1: This is a practical paper, describing five ways in which PEP could be implemented in a leprosy control program.

The list is very sensible, but many of the authors could probably have come up with the same list without having to do the study. So the authors need to show that their work had an element of rigour - that the results were not actually decided beforehand; this would be mainly demonstrated by a more detailed presentation of the results of the literature review and how those initial conclusions were refined through expert interviews.

*Thank you. We recognize the need to address this more clearly. We have done this by inserting additional text and a table (2) and figures (1 and 3).*

*The data extraction forms and interview topic lists have been uploaded as well.*

The words "unexperienced" and "capacitated" may or may not be real words in English but they are certainly inelegant and need to be changed.

*Thank you. This has been changed.*

Reviewer #2: Although not really an innovation, the tool resulting from the study offers a hands-on systematization to be applied on contacts tracing and so that favoring an early diagnosis and leprosy prevention.

*Thank you.*

Reviewer #3: I think this is very well written and very useful for the field of leprosy. It not just synthesizes much knowledge, but also continues to provide a valuable tool to support implementation. A great example of well-conducted applied research!

*Thank you.*

Reviewer #4: A strong point of the study is that the developed tool will have wide applicability in clinical practice, making it possible to generate a positive impact on the formulation of leprosy prevention policies.

*Thank you.*

Reviewer #5: The manuscript is to abstract and missing the important things that should describe, the author should include the important result in the manuscript not to ask the reader to open many links

*Thank you for your comments. By the adaptations made to the manuscript, we believe to have addressed your concerns.*

--------------------
