# Supplementary material for: Implementation approaches for leprosy prevention with single-dose rifampicin: A support tool for decision making
Source: PLoS Negl Trop Dis. 2022 Oct 17;16(10):e0010792. doi: 10.1371/journal.pntd.0010792 (PMC9612816; doi:10.1371/journal.pntd.0010792)
Supplement: Attachment — Submitted filename: 220708 Response to Reviewer comments.docx [file pntd.0010792.s005.docx]

**Reviewer's Responses to Questions**

An SDR-PEP implementation decision support tool

**Methods**

Reviewer #1: The Methodology has now been explained clearly.

Reviewer #3: The authors have answered all reviewer questions meticulously and clarified the methods section considerably.

Reviewer #4: Thank you for considering the suggested changes and justifying those that were not met. I have no further considerations on this topic.

Thank you.

Reviewer #5: -	In Phase 1. Literature review, please describe the search timeline of articles published (is it May to June 2020?) (page 7) because in the result the author mentioned that articles were published between 2005-2020 (page 10)

The first time period indicates when the search was performed: The search itself was done in May and June 2020. The second time period indicates when articles included in the search were published: the search was directed towards articles published in the period 2005 – 2020.

--------------------

**Results**

Reviewer #1: The Results are comprehensive and clearly presented.

Reviewer #3: Good improvements after reviewer comments.

Thank you.

Reviewer #4: Regarding the comment "In the results section – literature review, check the information regarding the correct number of articles excluded after reading the full text. The flowchart states that 55 articles were excluded, which is consistent with the final number included in the review (33), and the body of the text contains 52." performed in the review of the original submission, I believe that I may not have been clear about the divergence found. In this way, I reiterate the comment since I consider it important to review the data to avoid discrepancies in your text.

I did not refer to the greater number of articles used in the article's reference list in relation to those used in the literature review but to the divergence between the information on the number of articles excluded from the literature review in the full-text reading phase, which n = 55 in figure 2 and n = 52 in the text description in the 5th line of the topic Phase 1 a Literature review. Please check this data and make the necessary corrections.

Apologies for misunderstanding your comment. It is very clear now and a very valid point. We have corrected the typo in the text: 52 in changed into 55.

Reviewer #5: -	The result is more readable than the previous version.

-	Please do not put any comments, interpretations or references in the result, put them into the discussion (check page 14-27)

Thank you for your comment. However, it is not clear which parts of the results section the reviewer refers to that should be moved to the discussion. The results have quite some reference to literature (references) and expert opinion (comments and interpretations), because the results are derived from literature and interviews. We have not been able to identify parts that should move to the discussion section.

-	Where I and the reader can find the SDR-PEP decision support tool?

A link to the tool was provided in the first manuscript submitted. On request of the journal the tool was removed from the platform and send as an annex. We can provide a link to an online version of the tool again to insert in the published version of the article.

--------------------

**Conclusions**

Reviewer #1: Fully based on the Results presented.

Reviewer #3: All well-described and improved based on reviewer comments.

Reviewer #4: I consider that the new topic structure meets the proposed changes. I have no additional considerations on this topic.

Reviewer #5: Conclusion clear

Thank you.

--------------------

**Editorial and Data Presentation Modifications?**

Reviewer #1: None.

Reviewer #3: None suggested.

Reviewer #4: I consider that the new text structure meets the proposed changes. I have no additional considerations on this topic.

Reviewer #5: readable

Thank you.

--------------------

**Summary and General Comments**

Reviewer #1: The revised version is greatly improved.

Reviewer #3: I am happy with the improvements made based on all reviewer comments.

Reviewer #4: The main recommendations made in the original submission were met and/or justified by the authors. I reiterate just one comment referring to the results section. It does not detract from the merit of the manuscript, but I consider it important to be revised, thus avoiding divergence between figure 2 and the writing of the topic "Phase 1.a. Literature review."

Thank you for this comment – it has been addressed, see above.

I would like to point out the need to remove the hyperlink setting throughout the text, in places where it previously contained a link and was replaced by the reference to the tool (see SDR-PEP decision support tool). This change is necessary since even though the page has been removed, the text remains with the configuration to redirect to the page in question (<https://linkprotect.cudasvc.com/url?a=https%3a%2f%2fwww.leprosy-information.org%2fresource%2fdecision-tool-sdr-pep&c=E,1,M_c7C1cwgjD3mxNYX_llIOTZzBdyS4K2ZRJ6ysEpAEAOZtHcShF8adlj4I3v0i45T0m7zYJjGuJM5_SEAkYoZdo6YVVLJ_YqRkxdKBz7xrshrUR9Gg,,&typo=1> -implementation).

Thank you. The remaining hyperlinks have been removed. The link can be inserted again, if agreed by the Journal.

The changes made in this revision version made the text much clearer, which I believe will be useful in the adherence of professionals to using the proposed tool "Decision tool SDR-PEP implementation" since it has become easier to understand the process by which it was created, offering greater credibility to the tool.

Thank you.

Reviewer #5: -	The paper is more readable than the previous version, the author already put the additional table, and figures to make the article more readable.

-	It is still confusing between the result and discussion, the author mixed it

See response to this comment above.

-	Please provide the tool in the manuscript or link because it can not find in the link or in pdf

The tool can be made available through a link to an online version, or in a different way, as preferred by the Journal.
