# Supplementary material for: Implementation approaches for leprosy prevention with single-dose rifampicin: A support tool for decision making
Source: PLoS Negl Trop Dis. 2022 Oct 17;16(10):e0010792. doi: 10.1371/journal.pntd.0010792 (PMC9612816; doi:10.1371/journal.pntd.0010792)
Supplement: Attachment — Submitted filename: 220806 Response to reviewer comments.docx [file pntd.0010792.s006.docx]

**Response to Reviewers’ Questions**

An SDR-PEP implementation decision support tool

**Methods**

Reviewer #1: The Methods are clearly explained.

Reviewer #4: The methodology is clearly described.

Reviewer #5: -	In Phase 1. Literature review, please describe the search timeline of articles published (is it May to June 2020?) (page 7) because in the result the author mentioned that articles were published between 2005-2020 (page 10) --> please define in Phase 1: the time period the search was performed and the articles published

In line 174 the following phrase is added: *from articles published in the period 2005-2020*

In line 182 the time period of the search was already mentioned: *This search took place in May and June 2020*.

**Results**

Reviewer #1: The Results are clearly explained.

Reviewer #4: Thank you for considering the suggested changes. The results are now clearly described.

Reviewer #5: -	In Phase 2, the results are not from literature review but from user interview but author still put references in the results (page 26) so it’s mix between results from respondents and references.

In line 146-148 it is explained that the second phase uses data from the first phase: *The second phase was an iterative process where the data of the first phase was used and the usability and applicability of the decision support tool was evaluated with the intended users*

**Conclusions**

Reviewer #1: The Conclusions are fully justified.

Reviewer #4: The conclusion is clear and described based on the results.

Reviewer #5: -	Do not mix between result and discussion

In the previous comments from the reviewers, Reviewer #5 stated: Conclusion clear

No changes to the conclusion have been made, so it is not really clear what mixture between results and discussion Reviewer #5 wants to see addressed in the Conclusion.

**Editorial and Data Presentation Modifications?**

Reviewer #1: None needed.

Reviewer #4: None suggested.

Reviewer #5: none

**Summary and General Comments**

Reviewer #1: A comprehensive and much apprecited paper.

Reviewer #4: The authors made the suggested changes, which made this version much clearer and improved. I have no additional suggestions for changes to the article.

Reviewer #5: -	I hope the reader could easily access the tool to make comprehensive view. In the future the tool should be access widely to make useful.

As stated in the previous response to reviewers comments: a link to the tool was provided in the first manuscript submitted. On request of the journal the tool was removed from the platform and send as an annex. We can provide a link to an online version of the tool again to insert in the published version of the article.
